# Dermal γδ T Cells – What Have We Learned?

**DOI:** 10.1016/j.cellimm.2015.01.011

**Published:** 2015-01-28

**Authors:** Rebecca L. O’Brien, Willi K. Born

**Affiliations:** Dept. of Biomedical Research, National Jewish Health, 1400 Jackson St., Denver, CO 80206 and Dept. of Immunology and Microbiology, University of Colorado School of Medicine, 13001 E. 17th Place, Aurora, CO 80045

**Keywords:** gamma delta T cells, skin, dermis, epidermis, IL-17, CCR6, TCR

## Abstract

Over the last several years, a number of papers have called attention to a distinct population of γδ T cells preferentially found in the dermis of the skin of normal mice. These cells appear to play an important role in promoting the development of psoriasis, but also are critical for host resistance to particular pathogens. They are characterized by the expression of a limited subset of γδ T cell receptors and a strong propensity to secrete IL-17. Perhaps most importantly, humans appear to carry an equivalent dermal γδ T cell population, likewise biased to secrete IL-17 and also implicated as playing a pathogenic role in psoriasis. This review will attempt to summarize and reconcile recent findings concerning the dermal γδ T cells.

## 1. How were phenotypically distinct dermal γδ T cells identified?

Historically, one of the first characteristics of γδ T cells that distinguished them from classical αβ T cells was their relative abundance in certain anatomical sites, particularly in epithelial tissues. Moreover, the distribution of γδ T cells was found to be non-random in these tissues, and γδ T cells bearing certain T cell receptors (TCRs) predominated in distinct sites. One of the earliest examples of this was the discovery that nearly all T cells present in the epidermis of mice, known as dendritic epidermal T cells (DETC), are γδ T cells expressing identical TCRs, composed of Vγ5- and Vδ1-containing TCR chains that also carry identical or nearly identical junctional sequences [1] (note: the Tonegawa nomenclature for mouse Vγ chains will be used throughout this review [2]). Furthermore, cells bearing this canonical TCR were not found at any other location in the periphery mouse, and they evidently represent a specialized subset for the epidermis only. These cells are derived from thymic precursors generated only in the fetal/newborn stage of development [3], which home to the skin after exiting the thymus, and then persist throughout the life of the mouse by limited peripheral expansion. The invariant TCR that the DETC express is composed of germline-encoded components only. This feature is thought to be representative of the fetal development of these T cells, which occurs before deoxynucleotidyl transferase, the enzyme needed for N and P nucleotide additions to assembling antigen receptor genes terminal, is expressed [4].

Though the reason for a need for a particular T cell type in the epidermis having a predetermined specificity is still not understood, in the last few years it has become clear that another skin-associated γδ T cell population also exists, residing in the dermis rather than epidermis. The existence of these cells was first suggested by a finding from our laboratory in a study involving mice with collagen-induced arthritis. In this model, a disease with many of the same characteristics as human rheumatoid arthritis can be induced by intradermal injection of DBA/1 mice with a collagen/Complete Freund’s Adjuvant (CFA) emulsion. Upon examining the T cells present in the draining lymph nodes of mice with collagen-induced arthritis, we found a preferential increase in γδ T cells expressing a Vγ4Vδ4 TCR, and also showing a strong bias to secrete IL-17A. A more in-depth analysis revealed that these cells also express nearly invariant TCR junctions [5], which could either suggest the oligoclonal expansion of γδ T cells having a certain specificity, or that the cells represent, like DETC, a fetal-derived subset. This strong response by Vγ4Vδ4+ γδ cells was not dependent upon the mice developing arthritis, required CFA but not collagen in the immunizing inoculum [5], and depended upon immunization via the skin, by intradermal or subcutaneous inoculation, which we confirmed in a later study [6]. This requirement implied that the preferentially responding γδ T cells originate from the murine dermis. In fact, a paper by Kisielow et al. in 2008 reported that γδ T cells with these properties are present in the dermis, and showed that the skin-draining lymph nodes as well as the dermis contain a population of IL-17-biased predominantly Vγ4+ γδ T cells, representing about 20% of the dermal γδ T cells, also distinct in that they express high levels of the scavenger receptors Scart1 and Scart2. The Scart2-expressing γδ T cell subset was not detected among lymphocytes obtained from mesenteric lymph nodes or the spleen [7]. Soon after this publication, in a study involving an autoimmune uveitis model, a similar subset of γδ T cells was found to expand preferentially when cultured in the presence of IL-23; these cells likewise predominantly expressing Vγ4 and Vδ4 in their TCRs and were strongly biased to produce IL-17A [8]. The disease in this instance, which like collagen-induced arthritis is exacerbated by IL-17 [8, 9], was again provoked by a subcutaneous immunization, using an ocular antigen peptide emulsified in CF. As with collagen-induced arthritis, subcutaneous emulsified CFA alone was sufficient to elicit the response of these γδ T cells, again suggesting the possible dermal origin for the dominantly responding γδ T cell subset. Interestingly, the production of IL-17y these γδ T cells, which was elicited by culture with IL-23, required the presence of both γδ and αβ T cells, and the ability of the two cell types to make physical contact [8].

## 2. What are the characteristics of dermal γδ T cells?

In 2011, nearly simultaneous publications from three different laboratories [10–12] described the presence of a major γδ T cell subset present in the normal dermis having many characteristics in common with those we and others had noted among the γδ T cell subset responding preferentially following immunization with intradermal or subcutaneous CFA. In particular, the dermis-associated γδ T cells predominantly expressed IL-17 when stimulated with PMA/ionomycin [10–12], and about half expressed a Vγ4+ TCR [11, 12]. These dermal γδ T cells were shown to differ from the epidermal DETC subset in terms of the type of TCR they express (few were Vγ5+, whereas DETC are virtually all Vγ5+), in the amount of TCR present on their surfaces (DETC are TCR-bright whereas the dermal γδ T cell were found to be TCR-intermediate), and in the amount of the chemokine receptor CCR6 that they expressed (which is essentially absent on DETCs but abundant on dermal γδ T cells) [10]. The latter finding is interesting because CCR6, whose ligand CCL20 is expressed by epidermal keratinocytes, endothelial cells, and dendritic cells during skin inflammation, has been shown to play an important role in promoting the infiltration of activated T cells into the skin [13]; thus, CCR6 expression may imply that the dermal γδ T cells largely represent previously activated cells that have been recruited to the skin. An equally striking difference noted between the dermal γδ T cells and DETC was their motility: whereas DETC are sessile and remain in close contact with surrounding keratinocytes, the dermal γδ T cells were highly motile [10] (Table 1).

A number of other distinct properties of dermal γδ T cells were also noted. First, they were found to depend for their maintenance on IL-7 but not IL-15 [12], unlike DETC [14, 15] and splenic γδ T cells [16, 17] which depend upon both IL-7 and IL-15. Like the Vγ4Vδ4+ T cells elicited by intradermal CFA immunization [5], the dermal γδ T cells were found to carry additional cell surface molecules characteristic of T cells that have been pre-activated: virtually all were CD69-positive, CD44-high, and CD25-low [10, 12]. Second, as had been previously reported for naïve splenic RORγt-expressing IL-23R-positive γδ T cells [18,] naive dermal γδ T cells could be induced to proliferate and secrete IL-17 when cultured with cytokine as the only stimulant. This was evident either after 2 days in culture with IL-23 [11] or within 8 hours with IL-23 plus IL-1β [10]. Including ligands for TLRs or dectin in these cultures enhanced the effect of IL-23, perhaps by stimulating IL-1β production as well, because the ability to express IL-1β was found to be essential for this response [11]. Production by these cells of the other “IL-17 type” cytokines IL-17F and IL-22 as well as IL-17A was reported in two of these studies [10, 11], even though lymph node CFA-elicited Vγ4Vδ4+ cells when stimulated with PMA/ionomycin appeared to produce only IL-17A but not IL-17F or IL-22 [6]. When likewise stimulated with PMA/ionomycin, dermal γδ T cells produced large amounts of IL-17 plus an intermediate amount of TNFα and IL-22 [11]. Lymph node γδ T cells tested side-by-side also produced these same cytokines, but they also secreted IFNγ.

## 3. What is the immunological role of dermal γδ T cells?

Based on our findings with Vγ4Vδ4+ cells in the skin-draining lymph nodes of mice immunized intradermally with emulsified CFA, we anticipated that they would have an overall pro-inflammatory effect, and hence could play either a positive or negative role depending upon the disease in question. When we examined their role in mice with collagen-induced arthritis, inactivation/depletion of Vγ4+ cells by injection of a Vγ4-specific monoclonal antibody resulted in milder disease, indicating a disease-exacerbating role for this subset [5]. A negative role for dermal IL-17-producing γδ T cells has now been shown in several other studies involving induced dermatitis. When dermatitis was provoked by topical application of TPA, a phorbol ester, Scart1+ γδ T cells present in the skin increased in the dermis nearly 9-fold [19]. In two other studies [11, 20], mice were treated topically with Imiquimod cream, a TLR7 agonist that stimulates IL-23 production and thereby induces a psoriasis-like disease characterized by epidermal hyperplasia, parakeratosis, and dermal inflammatory infiltrates containing neutrophils and T cells [21]. Here, TCRδ−/− mice developed much milder disease than did wildtype mice. Because the IL-17 axis is known to be essential for psoriasis induction in these models [21], the relative resistance of the TCRδ−/− mice was attributed to their lack of dermal IL-17-producing γδ T cells rather than to their lack of DETC, since Imiquimod treatment induces dermal γδ T cells but not DETC to produce copious amounts of IL-17. In a similar psoriasis model in which the disease is induced by direct intradermal injection of IL-23, nearly identical results were obtained and CCR6+ dermal γδ T cells were again implicated as the source of pathogenic IL-17 [22]. Production of IL-23 by Langerhans cells has in fact now been shown to be required for the development of psoriasis in Imiquimod-treated mice, and for inducing IL-17 production from CCR6+ γδ T cells [23]. When skin from human psoriasis patients was examined, it was also found to contain elevated numbers of dermal γδ T cells compared to normal controls, increasing from an average of about 1% of the CD3+ cells in normal control samples to an average of 15% in psoriasis samples [11]. The psoriasis-associated human γδ T cells produced IL-17 when stimulated in culture, strongly implying that dermal γδ T cell subsets in mice and humans are in fact functional analogues of one another. Epidermal γδ T cells in humans also had similar functions as the murine DETC γδ T cells [24] although unlike mouse DETC they do not contain a TCR-invariant γδ T cell population.

Evidence for a positive role for dermal IL-17-producing γδ T cells in infectious disease was documented in mice infected intradermally with *Mycobacterium bovis*-BCG. Infection of these mice resulted in a rapid induction of IL-17 secretion among their dermal γδ (but not αβ) T cells, and this process appeared to be important in subsequent neutrophil recruitment, because neutrophil numbers in the skin were much reduced in TCRδ−/− mice compared to normal controls. Even more strikingly, the numbers of BCG bacteria subsequently detectable in the draining lymph nodes was greatly reduced in mice lacking γδ T cells compared to normal controls [12], implying that a major role of dermal γδ T cells may be to recruit, presumably via IL-17, neutrophil phagocytes into infected skin, in order to deliver antigens from the infectious agent to the central immune system.

Somewhat similar results were reported in an earlier paper, examining the role of γδ T cells in *Staphylococcus aureus* skin infections [25]. Here, TCRδ−/− mice developed strikingly larger lesions than normal controls when infected by intradermal injection of *S. aureus* (a three-fold difference), whereas mice lacking αβ T cells had lesions of similar size to the normal controls. Using bioluminescent *S. aureus* to track the infection, TCRδ−/− mice were also found to be inferior to both wildtype controls and αβ TCR-deficient mice in their ability to clear the infectious agent. Again, these results correlated with a decreased ability by TCRδ−/− mice to recruit neutrophils to the site of infection. They were likewise deficient in production of the neutrophil-mobilizing cytokines IL-17A and IL-17F, though not of IL-22, which also has this effect. However, the source of IL-17A and IL-17F in the wildtype mice in this study was found to be epidermal γδ T cells (DETC), rather than dermal γδ T cells. This is surprising because DETC produced little if any IL-17 in other studies [e.g. [10–12, 26]]. It seems possible, therefore, that this result reflects contamination of the purified DETC with dermal γδ T cells, as was in fact suggested by one laboratory [10]. However, if the FACS profile shown in this paper of purified DETC is typical (99.9% of the γδ TCR-positive cells were Vγ5-positive), not enough dermal γδ T cells were left to explain a strong IL-17 response. Much higher mRNA levels for IL-17A and IL-17F were also found in epidermal compared to dermal γδ T cell preparations from wildtype mice cutaneously infected with *S. aureus*, supporting the interpretation that DETC were indeed the source of IL-17 in this study. This study emphasized the ability of skin γδ T cells to produce IL-17 is critical for host resistance to *S. aureus*. Consistently, a recent report from the Havran laboratory showed that a subset of DETC are able to produce IL-17A following skin injury, and that these IL-17-producing cells play an important role in subsequent wound healing [27]. Therefore, at least under some circumstances, the IL-17-producing skin-derived γδ T cells appear to be DETC rather than cells of dermal origin, and their response can be important for the welfare of the host. It will be interesting to see in future experiments whether distinct stimuli induce IL-17 production by dermal vs. epidermal γδ T cells.

An important consequence of an IL-17 response by dermal γδ T cells is the enhancement of subsequent cell-mediated immunity. As shown earlier in an uveitis model, a response by IL-17-producing γδ T cells enhances the ensuing response of αβ Th17 cells stimulated by subcutaneous immunization [28], and although they are pathogenic in this model, Th17 cells have proven to be critical for host resistance to certain pathogens, particularly fungi and extracellular bacteria [reviewed in [29]]. Using mice immunized via intradermal injection with CFA, we found that pre-empting the Vγ4 response by pre-treating the mice with a Vγ4 inactivating/depleting monoclonal antibody depressed the ensuing αβ T cell response by nearly 2-fold [6]. Moreover, this also substantially reduced the numbers of αβ T cells biased to produce IFNγ, TNFα, and IL-17A. Consistently, Vγ4/6−/− mice, which cannot produce either Vγ4 or Vγ6 γδ T cells [30], when immunized intradermally with CFA showed a more than 2-fold reduction in CD4+ αβ T cells biased to produce IL-17A compared to wildtype controls [6]. These results suggest that the Vγ4Vδ4+ IL-17-producing γδ T cell subset, which responds preferentially in both the uveitis model and the CFA immunization system, promotes the concomitant development of proinflammatory αβ T cells, including Th17 CD4+ αβ T cells. This is consistent with results reported earlier by Sumaria et al., comparing wildtype to TCRδ−/− mice infected intradermally with *M. bovis*-BCG; the TCRδ−/− mice showed a nearly two-fold reduction in responding CD4+ αβ T cells in the draining lymph nodes compared to wildtype controls [12]. Interestingly, the converse of this finding, that IL-17-producing αβ T cells likewise promote the response of IL-17 producing γδ T cells, also may be true, because in in vitro culture experiments with purified αβ and γδ T cells from mice immunized subcutaneously with a uveitogenic peptide plus CFA, removal of either subtype from the culture greatly reduced IL-17 production elicited in response to the immunizing peptide [8]. Moreover, the Min laboratory has shown that even in naïve mice, Th17 CD4+ αβ T cells are needed to maintain IL-17-biased γδ T cells, via a process requiring TGFβ1 [31].

## 4. Is the IL-17 bias of dermal γδ T cells acquired in the thymus?

Unlike classical αβ T cells, γδ T cells emerge from the thymus already with a bias to produce either IL-17 or IFNγ, and those with an IFNγ bias were found to require thymic expression of a ligand for their TCR [32]. A recent publication from the Kang laboratory investigated transcription factors needed in the thymus to confer upon γδ T cells the ability to produce IL-17-type cytokines [33]. In agreement with a hypothesis put forth some years ago [32], conventional TCR signaling was not involved in the development of IL-17-producing Vγ4+ γδ T cells. Instead, the transcription factors SOX4 and SOX13, moderated by TCF1 and LEF1, were needed to induce a secondary transcription factor required by all cells that express IL-17, Rorc (encoding RORγt), as well as Blk (encoding B lymphocyte kinase), needed by γδ T cells for IL-17 production. SOX4 and SOX13 are expressed in developing γδ thymocytes at the immature stage (CD24-positive) but they subside at the mature stage (CD24-low). Various γδ T cell subsets present in SOX13−/−, SOX4−/−, and TCF1−/− mice were therefore examined for their ability to produce IL-17 when stimulated with PMA/ionomycin. SOX13−/− mice developed very few IL-17+ Vγ4+ cells, and most of them were also CCR6-negative and RORγt-negative, suggesting that induction of these “IL-17 signature” proteins is controlled by a common pathway. In contrast, Vγ6+ cells in the fetal thymus were reduced in SOX13−/− mice but had recovered to normal levels in adult mice, indicating that SOX13 is not required by the other major γδ T cell subset that produces IL-17, the Vγ6+ cells. SOX4−/− mice also lacked IL-17+ Vγ4+ cells, showing an even more severe depletion of these cells than the SOX13−/− mice, and although SOX4−/− mice also lacked RORγt expression in Vγ4+ cells, RORγt expression in αβ T cells was not affected, indicating that it is likely activated by a different pathway. Vγ6+ cells in SOX4−/− mice were somewhat reduced in number compared to wildtype but still developed and were able to secrete IL-17. In contrast, TCF1−/− mice had more than normal numbers of Vγ4 IL-17+ cells, and also produced considerably more Vγ1+ IL-17+ cells, which are usually quite rare. Inactivation of the TCF1 gene also decreased levels of CD27 (present on IFNγ+ γδ T cells) on γδ T cells even as immature thymocytes. Thus, TCF1 appears to have the opposite effect of SOX13 and SOX4, and instead acts to bias γδ T cells towards IFNγ rather than IL-17 production. This study went on to examine the role of ITK, needed for IL-17 production by Th17 αβ T cells, in IL-17 producing γδ T cells, and found that it is also required for Vγ4+ IL-17 production.

Finally, the Kang laboratory study tested whether or not the expressed TCR could be dictating the turn-on of factors that program a developing γδ T cell for IL-17 vs. IFNγ production. They found no evidence for a TCR role; instead, results from OP9-DL1 cultures suggested that a bias is already present in the precursor cells, because in this culture system, early thymic precursors (c-kit-positive thymocytes) give rise to CCR6+ CD27− IL-17-biased Vγ4 cells, whereas late precursors (c-kit negative DN3 thymocytes) give rise to CCR6− CD27+ IFNγ-biased Vγ1 and Vγ4+ cells. However, Vγ4+ Scart2+ cells, presumed precursors of the dermal Vγ4+ IL-17-producing population, were reported in the thymic DN3 population [7]; these precursors appear to be unable to complete their maturation in the OP9-DL1 culture system. Despite the Kang laboratory’s failure to find a role for the TCR during thymic development of these cells, a recent report from the Hayday laboratory suggested that the TCR is actually essential for the development of CD27-negative CD44-high γδ T cells, most of which are Vγ4+ or Vγ6+ IL-17-producers. Here, Zap-70 mutant mice, which produce low levels of a kinase essential for signaling through the TCR, were found to produce very few CD27-negative CD44-high γδ T cells. When mature, these γδ T cells in contrast to CD27+ IFN-γ producers normally were hyporesponsive to TCR signals, despite their very strong response to IL-23 plus IL-1β [34]. These results suggest that for Vγ4+ and Vγ6+ IL-17-producing γδ T cells, the TCR is in fact essential for thymic development, but may be unnecessary for their responses as mature cells, which are instead likely mediated through cytokine and perhaps other receptors.

## 5. Are dermal γδ T cells of fetal origin?

**Fig. 1:**
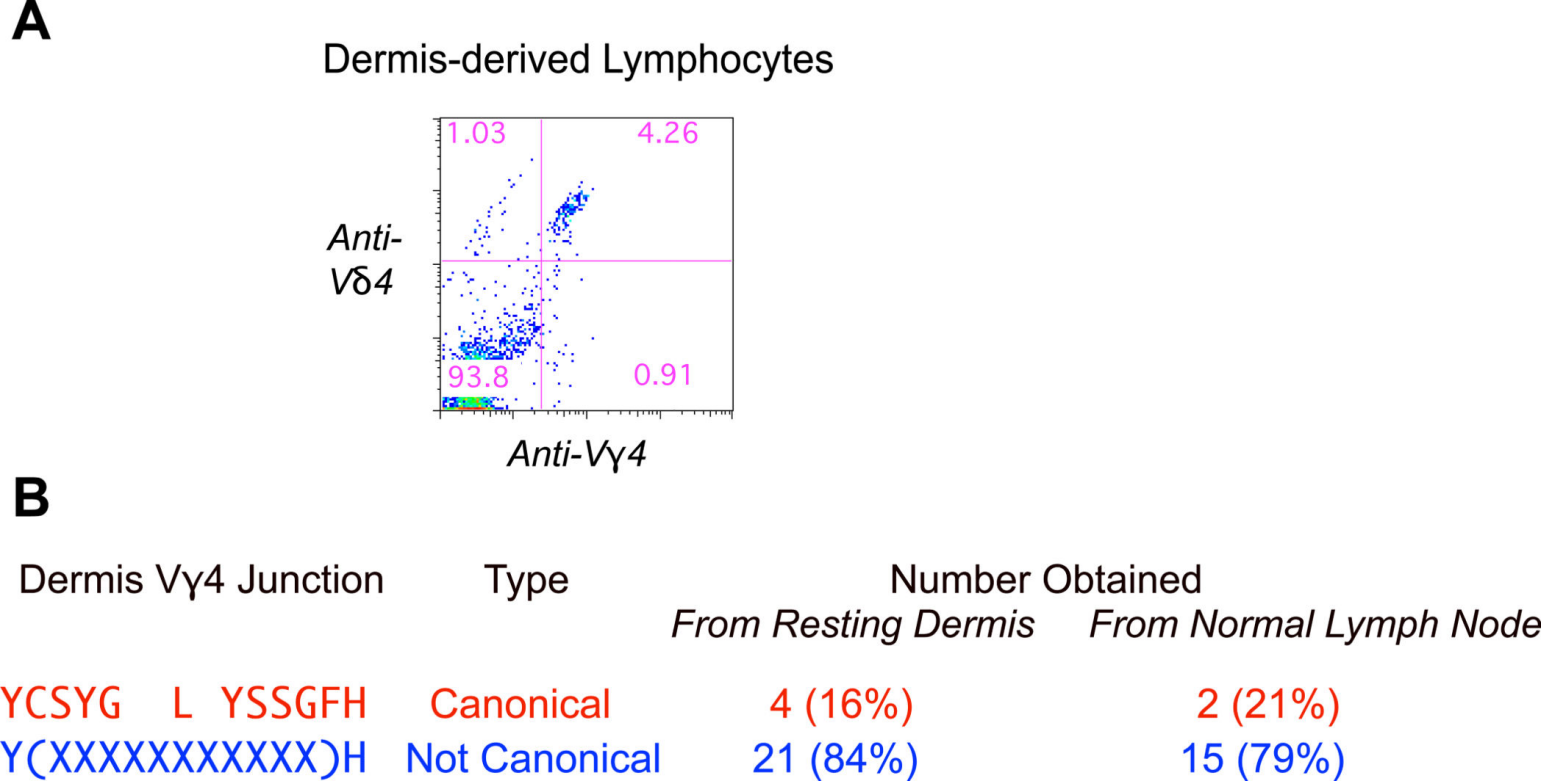
**A.** Flow cytometry profile of cells in the lymphocyte forward/side scatter gate prepared from ear dermis of C57BL/6J mice. **B.** Vγ4 junctional sequences were determined from cDNA generated from the dermis or lymph nodes of untreated C57BL/6J mice. PCR-amplified cDNAs were TA-cloned and sequences determined by conventional cycle sequencing.

In mice immunized intradermally with CFA, the majority of the responding Vγ4Vδ4+ γδ T cells subset carry TCRs whose junctions are very similar. [5, 6]. Invariant or nearly invariant TCRs, often referred to as “canonical” TCRs, are a trait of DETC [1], of Vγ6Vδ1+ γδ T cells [35] responding in several systems [reviewed in [36]], and of the iNKT-like Vγ1Vδ6.3+ cells [37]. The Vγ5Vδ1+ DETC and Vγ6Vδ1+ subsets are produced in the thymus of fetal or newborn mice and are no longer produced in adult mice; their TCR rearrangements take place under the control of a site-restricted recombination process that is only active during the fetal/newborn stage [4], but some selection for cells whose TCRs carry these particular sequences evidently also occurs [38]. The iNKT-like Vγ1Vδ6.3+ cells, which also have canonical TCR junctions, in contrast appear to be exclusively selected at the cellular level, as no evidence was found for orchestrated gene rearrangements in these cells [39]. The TCR junctions of the IL-17-producing Vγ4Vδ4+ cells elicited by intradermal CFA immunization, despite having nearly identical protein sequences, use multiple codons to encode the junctional amino acids [5], suggesting that the cells bearing TCRs with these particular junctions are in some way selected. Because the other three TCR-invariant γδ T cell types have all been shown to develop exclusively or mainly during fetal or newborn life [40, 41], the IL-17 producing Vγ4Vδ4+ cells with canonical TCR junctions could likewise be of fetal and/or newborn origin, and indeed, a recent paper from the Prinz laboratory presented evidence that γδ T cells that develop an IL-17 bias only develop in the thymus during fetal life [42]. Whether the dermal Vγ4Vδ4+ γδ T cells typically express the canonical TCR has not been examined so far. We therefore addressed this issue by sequencing Vγ4-containing transcripts from the dermis of normal C57BL/6 mice. As shown in Fig. 1, although many Vγ4+ dermal γδ T co-express Vδ4 (Fig 1A), most dermal Vγ4 TCR transcripts had a non-canonical junction (Fig. 1B); in fact, the percentage having canonical Vγ4 junctions (16% in this study) was even lower than was found in the lymph nodes of naïve mice of the same strain (21%). This suggests that the Vγ4Vδ4+ γδ T cells that preferentially respond following intradermal CFA immunization usually have canonical TCR sequences because they are by their TCR specificity selected to respond, rather than because they are preferentially generated and/or pre-selected during thymic development. Therefore, whereas dermal IL-17-producing Vγ4Vδ4+ cells may still be of fetal origin, their TCRs largely do not conform to a canonical sequence and thus, they do not appear to represent a subset whose specificity has been pre-selected in the thymus.

Vγ5Vδ1+ DETC, being generated only in the fetal thymus, cannot be reconstituted by the adoptive transfer of adult bone marrow precursors, and it was found early on that DETC are also relatively resistant to irradiation [43]. Two different laboratories have reported that dermal γδ T cells are also radio-resistant [10, 12], emphasizing their similarity to DETC and consistent with the idea that these cells might also be of fetal or newborn origin. Furthermore, like DETC, the dermal γδ T cells undergo local homeostatic proliferation in the dermis, and in bone marrow-reconstituted irradiated mice, they cannot be re-seeded from circulating γδ T cells, even though dermal αβ T cells are readily reconstituted from circulating precursors [12]. Including in the adoptive transfer fetal thymocytes along with bone marrow allows for successful reconstitution of dermal IL-17-biased γδ T cells in irradiated hosts [10]. In neither study was the Vγ or Vδ makeup of the TCRs of the reconstituted cells examined, however. A recent paper from the Yan laboratory reported the additional observation that although many dermal γδ T cells are indeed Vγ4+, an approximately equal number instead expresses Vγ6Vδ1 [44]. This subset, most of whose members carry a canonical TCR, preferentially responds in a wide variety of disease models [45], and these cells are well-known as producers of IL-17. This result was rather surprising since the same laboratory had barely detected the Vγ6Vδ1+ subset among dermal γδ T cells in an earlier study [11]. They did not discuss the reason for this discrepancy, but perhaps the antibody that identifies Vγ6Vδ1+ cells, 17D1, was initially not tested properly [46], and two other laboratories since have also reported that Vγ6+ cells comprise a substantial portion of the mouse dermal γδ T cells [33, 47]. Although they represent virtually all of the dermal γδ T cells in newborn mice, Vγ6Vδ1+ cells comprise only about 40% of the γδ T cells in adult dermis, as the Vγ4+ component gradually increases in the dermis over time. In reconstitution studies, the Yan laboratory confirmed that Vγ6+ cells are radio-resistant and require a source of fetal thymocytes for their reconstitution, whereas Vγ4 cells are radio-sensitive but can be reconstituted with bone marrow [44]. This latter point disagrees with the Cyster laboratory’s original finding that dermal γδ T cells cannot be reconstituted with bone marrow [10], although later this laboratory was able to find bone marrow reconstitution of Vγ4+ dermal cells after a longer incubation period [47]. The Yan laboratory went on to show that thymic Vγ4+ cells cannot directly reconstitute the dermis, unlike Vγ6+ thymocytes, but instead must first go to the periphery and mature, and they only migrate to the dermis when they acquire skin-homing properties such as turning on expression of CCR6. They speculated that CCR6 expression is crucial for homing to the dermis, because the dermal γδ T cells are all CCR6+, and whereas thymic Vγ4+ cells are CCR6-negative, the Vγ6+ thymocytes are already CCR6+. Interestingly, the Vγ6+ cells are able to outcompete Vγ4 cells for colonization of the dermis, perhaps because the Vγ4+ cells need this extra peripheral induction step before they do so [44].

## 6. Do both Vγ4+ and Vγ6+ dermal IL-17 producing γδ T cells promote psoriasis?

When SOX4−/− mice were examined for their ability to generate γδ TCR+ thymocytes biased to produce IL-17, it was also noted that whereas adult SOX4−/− mice carry very few Vγ4+ cells in the dermis, they contain fairly normal numbers of dermal Vγ6Vδ1+ cells. However, these SOX4−/− mice developed only mild skin inflammation when treated with Imiquimod, suggesting that Vγ4+ cells instead are the dermal γδ T cell subset that promotes psoriasis [33]. SJL mice with a spontaneous SOX13 mutation were also examined, which fail to develop Vγ4+ dermal cells but can still generate Vγ6+ dermal cells. These mice developed much less severe psoriasis when treated with Imiquimod than did wildtype SJLs [47], suggesting again that the Vγ4+ dermal γδ T cells are mainly responsible for the development of psoriasis. The Yan laboratory confirmed these studies by showing that whereas Vγ6 cells can induce some degree of skin inflammation, Vγ4 cells are more pathogenic and the primary IL-17 producers in the Imiquimod model. Here, they generated mice having mainly dermal Vγ6+ cells but not Vγ4+ γδ T cells by reconstituting irradiated TCRδ−/− hosts with purified Vγ6+ thymocytes plus bone marrow from TCRδ−/− donors, as well as mice having mainly Vγ4+ but not Vγ6+ γδ T cells by reconstituting the same hosts with wildtype bone marrow only. Both groups developed approximately equivalent and severe psoriasis symptoms after Imiquimod treatment, even though those with Vγ4+ dermal γδ T cells only had considerably less dermal γδ T cells overall than those having Vγ6+ dermal γδ T cells only. Imiquimod proved to be a better inducer of IL-17 in the Vγ4+ than in Vγ6+ dermal cells, and induced more proliferation of Vγ4+ than of Vγ6+ cells as well. Moreover, IL-23, induced by Imiquimod, when given with IL-1β stimulated more IL-17 production from the Vγ4+ than from Vγ6+ cells [44].

## 7. How do the dermal γδ T cells traffic?

The αβ T cells found in skin are mostly represented by tissue-resident CD8 effector memory cells, which develop following an infection, are found mainly in the epidermis and persist for long periods, and CD4+ cells, found mainly in the dermis. The dermal CD4+ cells include Tregs, which in uninflamed skin are largely non-motile, as well as both effector and naive CD4+ cells, that in contrast are highly motile and appear to migrate rapidly through resting skin in search of potential antigen [reviewed in [48]]. The CD4+ cells migrate from lymphatic vessels into the dermis by virtue of expressing skin homing receptors, such as CLA, and if they fail to encounter their antigen, then exit back to the lymphatics in a CCR7-dependent manner [49]. In contrast, though dermal γδ T cells like CD4+ αβ T cells are motile, they move at a slower speed than do the CD4+ T cells [10, 12]. However, when inflammation was induced in the skin, Scart1+ γδ T cells were found in increased numbers in the skin and could be found in both the dermis and epidermis [19]. CCR6, expressed by both Vγ4 and Vγ6 γδ T cells, is needed to allow dermal γδ T cells to move into the inflamed epidermis [22]. This step appears to be mediated by induced expression of the CCR6 ligand CXCL20, which keratinocytes and dendritic cells in the epidermis express in the presence of IL-23 [22]. Findings from the Weniger laboratory showed that dermal γδ T cells do not express CCR7, and they suggested that these cells therefore do not exit the skin under resting conditions [12]. However, the Cyster laboratory was able to demonstrate movement of dermal γδ T cells to the lymph node, and of lymph node γδ T cells into the dermis and then epidermis, using transgenic mice expressing a photo-convertible protein that turns from green to red following exposure to light of the correct wavelength [47]. A low level of movement of light-exposed skin γδ T cells into the draining lymph nodes was evident under resting conditions, whereas inducing skin inflammation by treatment with Imiquimod caused this number to increase. A concomitant selective expansion of Vγ4Vδ4+ IL-17+ cells in the relevant draining lymph nodes was also noted. Curiously, CCR6 was low or absent on many of these lymph node cells, which may suggest that CCR6 downregulation promotes their movement from the skin into the lymphatics. Adoptive transfer of draining lymph node cells from wildtype Imiquimod-treated mice into SOX13 mutant mice, which lack Vγ4+ IL-17+ cells, followed by treatment of the recipients with Imiquimod, resulted in infiltration of donor-derived cells in the inflamed skin of which a majority were Vγ4+ IL-17-producing cells [47]. Thus, it seems that the Vγ4+ dermal cells are able to migrate efficiently from the lymph node into inflamed skin. Whether the Vγ6+ dermal γδ T cells can also move into the lymph nodes when skin inflammation is induced has yet to be directly examined, but the results from the Cyster laboratory suggest that if so, it happens to a much smaller degree than for the Vγ4+ cells [47]. In a separate study, induction of skin inflammation by applying Imiquimod topically to the epidermis promoted the preferential expansion of Vγ4+ IL-17 producing cells, but did not specifically stimulate expansion of the dermal Vγ6+ cells, although some proliferation of Vγ6+ cells was evident by Ki-67 staining and they showed some increase in production IL-17A, though to a lesser than Vγ4+ cells [44]. Likewise, induction of inflammation by intradermal injection of CFA resulted in the preferential increase of Vδ4Vγ4+ IL-17 producing cells in the draining lymph nodes, but no proportionate increase in Vγ6+ cells was seen (Roark et al., unpublished results). Therefore, there appear to be differences in the trafficking of the Vγ4+ vs. Vγ6+ dermal γδ T cells, but whether this is due to lesser activation of the Vγ6+ cells in the models examined, as compared to the Vγ4+ cells, remains to be seen.

## 8. What is known about human dermal γδ T lymphocytes?

Several parallels indicate that in humans as well as in mice, an IL-17-biased γδ T cell population resides in the dermis and plays an important role in exacerbating psoriatic dermatitis. When skin biopsies from psoriasis patients were compared with those from normal controls, many more CD3+ cells were found in the psoriatic skin, and a large proportion of those expressed a γδ TCR: in fact, on average more than 40% of the CD3+ cells were γδ TCR+ compared to less than 15% in normal controls. When cultured with IL-23 (without IL-1β), on average about 10% of the γδ T cells from psoriatic skin were induced to secrete IL-17, whereas in cultures from normal skin, IL-17 production was almost undetectable [11]. In a separate study, CLA+ CCR6+ γδ TCR+ cells in human blood were examined, and were found to be substantially reduced in the blood of psoriasis patients. In this study as well, γδ T cells were found to be elevated in the skin lesions of psoriasis patients, and most of them expressed the Vγ9Vδ2+ TCR associated with “phosphoantigen” reactivity, normally the most prevalent γδ TCR+ population in the blood. A low percentage of Vγ9Vδ2+ cells in the blood also correlated well with disease severity, suggesting that these γδ T cells have exited the blood to enter the skin during the development of psoriasis, where they play a pathogenic role [50].

These reports came as something of a surprise because formerly, in the normal human dermis, Vδ1+ γδ T cells were found to predominate and Vδ2+ γδ T cells were rare [51]. The dermal Vδ1+ cells were found to express the skin-homing receptors CLA and CCR8, and when stimulated to produce TNFα and IFNγ (although IL-17 was not tested); they also have cytolytic capacity. Moreover, Vγ9Vδ2+ cells biased to produce IL-17 are quite rare at least in human peripheral blood [52], although there is some controversy on this point, particularly with regard to tuberculosis patients [53]. Thus, it appears that humans differ from mice in presence of dermal γδ T cells in resting skin: normal human dermis contains few if any γδ T cells capable of inducing psoriasis or related skin diseases, whereas in mice, two γδ T cell subsets having the potential to induce psoriatic disease normally reside in the resting dermis. Whether human dermal IL-17-producing γδ T cells can also be beneficial is not clear. However, individuals whose ability to produce IL-17A or IL-17F is impaired have a strong tendency to develop both candidiasis and staphylococcal infections in the skin or mucosae [reviewed in [54]]. Considering the high levels of IL-17 that these human dermal γδ T cells produce compared to dermal αβ T cells, they are likely to be key in host resistance to certain fungal and extracellular bacterial pathogens directly at the site of infection.

## 9. Do the unique characteristics of dermal γδ T cells suggest how they may function?

Both the Vγ4Vδ4+ IL-17 producing cells found in the draining lymph nodes of CFA-immunized mice [5] as well as the Vγ4+ and Vγ6+ γδ T cells found in the dermis [12, 44] typically express cell surface molecules associated with T cell activation (e.g. CD69+, CD44-high, CD62L-low), and dermal γδ T cells also produce IL-17 when stimulated with cytokine alone [10, 44], suggesting that these cells have been pre-activated. The expression of CCR6 by the dermal γδ T cells also suggests that the cells are already partially activated, especially in the case of the Vγ4+ cells, which exit the thymus as CCR6-negative cells and evidently must be induced to express CCR6 before they can migrate into the dermis [44]. In this way, dermal γδ T cells may be similar to the DETC in the epidermis, because in resting skin, the TCRs of DETC are pre-polarized: they form aggregates containing phosphotyrosine (PALPs) that are located almost exclusively along the squamous keratinocyte junctions [55]. This could indicate that DETC TCRs are constitutively bound to a self-ligand expressed by keratinocytes, and that for this reason their TCRs have already been organized into an immunological synapse, such that the cells require only a second signal (e.g. a cytokine and/or co-receptor), or perhaps a shift in the balance of activating vs. inhibiting co-receptors, to become fully activated. If so, it would be easy to imagine that the TCRs of γδ T cells in the dermis are, like DETC, already bound to a self-ligand and organized into an immunlogical synapse, and can therefore be induced to secrete IL-17 with only one additional signal, such as IL-23.

In a recent publication from the Chien laboratory, peripheral γδ T cells that recognize phycoerythrin were found to respond to alum immunization with this antigen by producing IL-17, acquiring classical activation markers (they became CD44-high and CD62L-low), and by altering their chemokine receptors [56]. They did not produce IL-17 until they had been activated with the antigen for their TCRs, and only after antigen activation did they begin to express receptors for IL-23 and IL-1. These observations support the notion that dermal γδ T cells, which constitutively express IL-23R [11], have indeed been pre-activated by antigen and await only a second signal to become fully activated effectors secreting IL-17.

## 10. Why are dermal γδ T cells needed?

The Havran laboratory’s finding that DETC under some circumstance also produce IL-17 [27] begs the question of why, in mice at least, special dermal γδ T cell subsets are needed, since DETC can also provide a source of this critical cytokine. If indeed the dermal γδ T cells are preactivated, the answer might lie in the identity of the antigens recognized by their TCRs. Perhaps, akin to intestinal γδ T cells [57], microbes that colonize the skin are responsible for the activation and accumulation of the Vγ4Vδ4+ and Vγ6Vδ1+ cells comprising most of the γδ T cells in the dermis.

## Figures and Tables

**Table 1:** Comparison of Characteristics of Dermal, Epidermal, and Splenic γδ T cells

Characteristic	Dermal γδ T cells	Epidermal γδ T cells (DETC)	Splenic γδ T cells
TCR composition	30–50% Vδ4Vγ4+ 50–60% Vδ1Vγ6+[33, 44]	98%+ Vδ1Vγ5+, canonical TCR [1]	Various
TCR levels	Intermediate [10, 11]	High	Intermediate
Chemokine receptors	CCR6+[10, 11]	CCR6−	CCR6− (~90%) [10]
Activation state	Pre-activated? CD69+ CD44-hi CD62L-lo [12, 44]	Pre-activated? CD69+ CD44-hi CD62L-lo TCR PALPs [55]	Most naïve ~75% CD69− CD44-lo CD62L-hi [12]
Radio-resistant	Vγ6: Radio-resistant [44] Vγ4: Radio-sensitive [10, 44]	Radio-resistant	Radio-sensitive
Requirements for development	Vγ6: fetal/newborn thymus [44] Vγ4: bone marrow sufficient [44]	Fetal/newborn thymus [3]	Bone marrow sufficient for most
Morphology	Motile [10]	Dendritic, sessile [1]	Motile
Unique cell surface markers	Scart1 and Scart2 [7]		
Cytokines produced	IL-17A, IL-22, IL-17F [10–12]	KGF, IFNγ, IL-2 [58]	IFNγ, IL-17 [32]
Cytokines required for maintenance	IL-7 [12]	IL-7 and IL-15 [14, 15]	IL-7 and IL-15 [17]
Needed in thymus for development	SOX4 and SOX13 for Vγ4+ [33]	Skint-1 [59]	PLZF (for some Vγ1+) [60]

## Highlights

- Most mouse dermal γδ T cells are IL-17+, CCR6+, motile, and Vγ4Vδ4+ or Vγ6Vδ1+
- They are pathogenic in psoriasis, but enhance protection against some pathogens
- They also enhance CD4+ αβ T cell-mediated immunity
- Human dermal γδ T cells are analogous in producing IL-17 and exacerbating psoriasis
- Responses by dermal γδ T cells can be triggered without the addition of antigen

## Abbreviations used

TCR: T cell receptor
DETC: dendritic epidermal T cells
CFA: Complete Freund’s Adjuvant
